# The effectiveness and safety of sanfu acupoint herbal patching for treating allergic rhinitis: A protocol for systematic review and meta-analysis

**DOI:** 10.1097/MD.0000000000024121

**Published:** 2021-01-08

**Authors:** Qiaochu Zhu, Zhongyu Zhou, Dan Wei, Yang Jiao, Yangpu Zhang, Xiaohui Tian, Yan Wang, Fu Dong, Aiqun Song

**Affiliations:** a Hubei University of Chinese Medicine; b Hubei Provincial Hospital of Chinese Medicine; c Hubei Province Academy of Traditional Chinese Medicine; d Hubei Provincial Hospital of Integrated Chinese and Western Medicine, Wuhan, China.

**Keywords:** allergic rhinitis, meta-analysis, sanfu acupoint herbal patching

## Abstract

**Background:**

Allergic rhinitis (AR) is one of the most common chronic disease of the nasal mucosa globally. Several clinical studies have shown that sanfu acupoint herbal patching (SAHP) has obvious advantages in treating AR. Therefore, the purpose of this systematic review is to evaluate the effectiveness and safety of SAHP for treating AR.

**Method:**

The following 9 electronic databases will be searched from January 2010 to October 2020: PubMed/Medline, Web of Science, Cochrane Library, EMBASE, China National Knowledge Infrastructure, VIP Database, WANFANG Database, China Biology Medicine disc. The selection of the studies and the extraction of the data are independently completed by 2 reviewers. The qualities of the studies are evaluated by Cochrane risk-of-bias tool. The main outcome of included studies is total effective rate. Secondary outcomes are Total Nasal Symptom Score, recurrence rate, Rhinitis Quality of Life Questionnaire, adverse events and laboratory indicators: serum immunoglobulin E (IgE). And the STATA 14.0 software will be implemented for data synthesis and meta-analysis.

**Results:**

The review is ongoing, no results can be reported.

**Conclusions:**

The systematic review will provide a better option for patients to treat AR.

**Registration number:**

INPLASY2020100101.

## 1 Introduction

Allergic rhinitis (AR) is defined as a non-infectious inflammatory disease of the nasal mucosa. The mechanism of this disease is due to immunoglobulin E (IgE)-mediated mast cell degranulation and mediator release, which can cause rapid response to allergens, leading to a series of clinical symptoms,^[1]^ such as nasal congestion, nasal itching, sneezing, and runny nose.^[2]^ According to epidemiological surveys, the prevalence of AR has risen rapidly in recent years. It has been calculated that 10% to 30% of the world population suffered from this disease approximately.^[3]^ A study which target the number of AR patients and the burden of this disease in America, Latin America and Asia-Pacific shows that the prevalence of American adults is 14% and children account for 13%, Latin American adults about 7%, Asia-Pacific adults about 9%.^[4]^ AR is known as a chronic disease, hence, quality of patients life has been disturbed impressively with its recurrence more and more frequently.^[5]^ Besides the original symptoms of AR have been mentioned above, quality of patients life has been weaken in the following aspects: physical function decreasing, social difficulties, sleep disturbance, fatigue during the day, lethargy, irritability, depression, lack of learning and memory.^[6]^ In the meanwhile, the economic burden of AR patients is substantial, the total cost of treatment reaches $3.4 billion per year and the main components of the cost are prescription drugs and outpatient visits.^[7]^ Patients always easily neglect the comorbidities of AR, such as headache, allergic conjunctivitis, otitis media, sinusitis, cough, asthma, which also exert a huge impact on their health.^[6]^ The relationship between AR and asthma should be taken into consideration because 78% of asthma patients have AR according to the study,^[8]^ which enlightens us that AR may be a high risk factor for asthma. Considering so many comorbidities, AR patients are required to pay more attention to the treatment of original disease and prevention of comorbidities so that we can reduce indirect economic investment.

Nowadays, oral H1 antihistamines and intranasal corticosteroids spray are widely acknowledged as the mainstream therapy for treating AR.^[9,10]^ These 2 major classes of drugs block inflammatory pathways to relieve nasal symptoms. Intranasal corticosteroids and H1 antihistamines are treated as first-line treatments with its own efficacy.^[11]^ The mechanism of Histamine is inhibiting basophils and mast cells from releasing, thereby reducing the binding to various histamine receptors of glandular, neurogenic and vascular target cells, which can decrease sensory nerve stimulation and subsequent parasympathetic activity.^[12]^ However, the disadvantages of this therapy has become obvious. With the recurrence of symptoms, the efficacy of the drugs will decrease; there is no doubt that the inhibition of symptoms requires more money and more dosage of drugs as well.^[6]^

Consequently, complementary and alternative therapy becomes a better option to treat AR,^[13]^ particularly traditional Chinese medicine (TCM).^[14–16]^ According to theory of TCM, sanfu refers to the 3 specific periods of the Chinese lunar year from July to August, each fu period includes 10 days.^[17]^ Sanfu period is characterized as the hottest temperature and the strongest power of Yang, which is defined as a metaphysical energy with a nature of hotness inside of the body to protect body from illness.^[18]^ Sanfu acupoint herbal patching (SAHP), as one of complementary and alternative therapy derived from Qing dynasty (1636–1912), also has good effect in treating AR. SAHP is a herbal pad that contains powdered Chinese herbals and is being used to stick to specific acupoints during sanfu period ^[19]^ The purpose of this treatment is to utilize the comprehensive effect of herbal medicine absorbed by skin, acupoint stimulation and time effect so that therapeutic effects can be produced.^[18]^ The efficacy of SAHP has been reported by a great number of randomized controlled trails (RCTs).^[20–24]^ However, no systematic review or meta-analysis about SAHP for treating AR has been reported yet. In this review, we aim to evaluate the effectiveness and safety of SAHP for treating AR.

## 2 Methods

### 2.1 Study registration

The protocol of this meta-analysis has been registered in INPLASY (https://inplasy.com/) with ID INPLASY 2020100101. Moreover, the protocol will comply with the Cochrane Handbook for Systematic Reviews and Meta-Analysis Protocol guidelines.^[25]^ Ethical approval can be skipped because this is a systematic literature research. Any changes will be explained if necessary.

### 2.2 Eligibility criteria

#### 2.2.1 Types of studies

All of RCTs published in English and Chinese about SAHP for treating AR will be included.

#### 2.2.2 Types of participants

Patients diagnosed with AR^[5]^ can be included into study regardless of nation, gender, race, courses of disease.

#### 2.2.3 Type of interventions

SAHP (No restriction on herbal medicine composition, course of disease, selection of acupoints, etc.) or SAHP plus other therapy (such as oral Chinese medicine, moxibustion, acupuncture, placebo, western medicine) as control group. However, some of RCTs with SAHP as an adjunctive treatment will be excluded.

#### 2.2.4 Types of comparisons

The intervention of comparator could be no treatment, placebo, sham acupoint herbal patching, or other therapy which involved in the control group. The possibilities of therapy combination will be listed:

1. SAHP vs no treatment.
2. SAHP vs other common therapy.
3. SAHP + other common therapy vs other common therapy.
4. SAHP vs placebo or sham acupoint herbal patching.

#### 2.2.5 Outcomes

##### 2.2.5.1 Primary outcomes

Total effective rate will be defined as main outcome in this meta-analysis.

##### 2.2.5.2 Secondary outcomes

TNSS (Total Nasal Symptom Score), recurrence rate, RQLQ (Rhinitis Quality of Life Questionnaire), adverse events, laboratory indicators: IgE will be used as secondary outcomes.

### 2.3 Exclusion criteria

1. Patients with AR accompany by other diseases.
2. Systematic review or meta-analysis, case report, animal experiments, conference papers will be excluded.
3. Repeated publications and missing data researches will be excluded.

### 2.4 Search methods for identification of studies

#### 2.4.1 Electronic searches

Owing to popularity of SAHP and high-quality researches published in recent 10 years, we will search the following databases from January 2010 to October 2020: PubMed, Web of Science, Cochrane Library, EMBASE, China National Knowledge Infrastructure, VIP Database, WANFANG Database, China Biology Medicine disc. The languages of included studies are restricted in English and Chinese. The keywords contain: “allergic rhinitis,” “sanfu acupoint herbal patching,” “randomized controlled trail”. In addition, clinical researches which are not completed will be searched on the mainstream registries such as Chinese Clinical Trail Registry and Clinical Trials.gov trials registry.

#### 2.4.2 Search strategy

The search strategy of PubMed is shown in Table 1, and other databases will be the same way to be searched.

**Table 1:** Pubmed will be searched from January 2015 to October 2020.

Search number	Query
#1	“Rhinitis, Allergic” [Mesh]
#2	((Allergic Rhinitides [Title/Abstract]) OR (Rhinitides, Allergic [Title/Abstract])) OR (Allergic Rhinitis [Title/Abstract])
#3	Rhinitis, Allergic, Seasonal [MeSH Terms]
#4	((((((((((((((Seasonal Allergic Rhinitis [Title/Abstract]) OR (Allergic Rhinitides, Seasonal [Title/Abstract])) OR (Allergic Rhinitis, Seasonal [Title/Abstract])) OR (Rhinitides, Seasonal Allergic [Title/Abstract])) OR (Rhinitis, Seasonal Allergic [Title/Abstract])) OR (Seasonal Allergic Rhinitides [Title/Abstract])) OR (Pollen Allergy [Title/Abstract])) OR (Allergies, Pollen [Title/Abstract])) OR (Allergy, Pollen [Title/Abstract])) OR (Pollen Allergies [Title/Abstract])) OR (Pollinosis [Title/Abstract])) OR (Pollinoses [Title/Abstract])) OR (Hay Fever [Title/Abstract])) OR (Fever, Hay [Title/Abstract])) OR (Hayfever [Title/Abstract])
#5	#1 OR #2 OR #3 OR #4
#6	acupuncture points [MeSH Terms]
#7	(((((((((Acupuncture Point [Title/Abstract]) OR (acupoint [Title/Abstract])) OR (Acupoints [Title/Abstract])) OR (sanfu moxibustion [Title/Abstract])) OR (Sanfu acupoint herbal patching [Title/Abstract])) OR (sanfu herbal patch [Title/Abstract])) OR (acupoint sticking [Title/Abstract])) OR (summer acupoint herbal patching [Title/Abstract])) OR (Sanfujiu [Title/Abstract])) OR (Sanfutie [Title/Abstract])
#8	#6 OR #7
#9	randomized controlled trial [Publication Type] OR (randomized [Title/Abstract] AND controlled [Title/Abstract] AND trial [Title/Abstract])
#10	#5 AND #8 AND #9

### 2.5 Data collections and analysis

#### 2.5.1 Selection of studies

**Figure 1:**
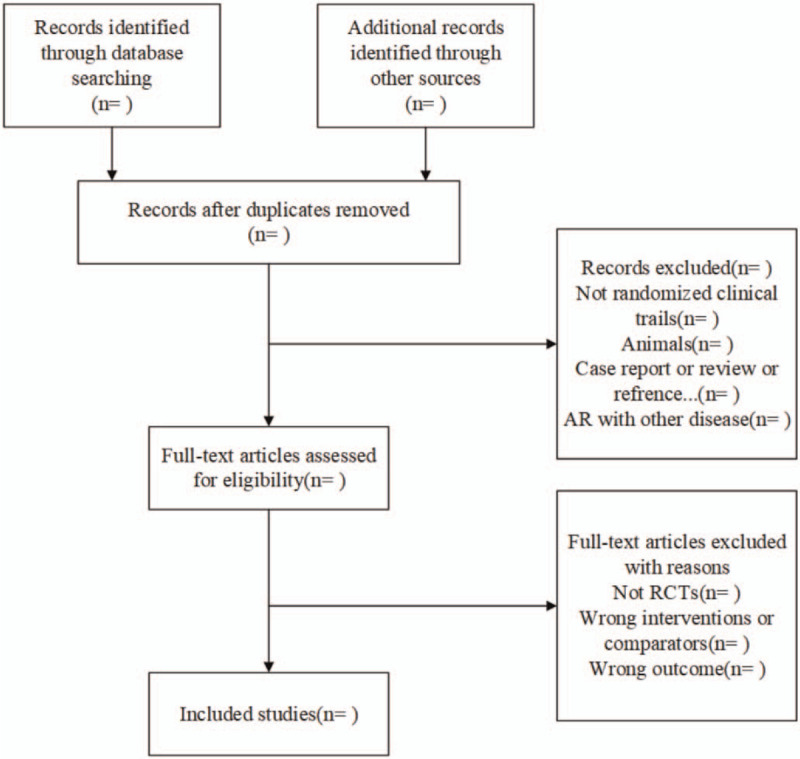
Flow diagram of the study selection process.

All retrieved studies will be managed with Note Express 3.0. Firstly, 2 reviewers (Xiaohui Tian and Qiaochu Zhu) will exclude some studies that are not in accordance with the inclusion criteria by reading titles and abstracts of the articles independently, then they will download included studies and read full text in order to check the studies whether they are available for inclusion criteria. Besides, if there is a dispute during the process of systematic review, consensus will be reached by discussion with the third reviewer (Aiqun Song). The process of screening will be shown in Figure 1.

#### 2.5.2 Data extraction and management

Two reviewers (Yan Wang and Yangpu Zhang) will extract data of included studies independently, and the following items will be extracted:

1. The first author, publication year, number of each group, average age.
2. Courses of disease, interventions, comparisons, courses of treatment.
3. Main outcome, secondary outcomes, blinding, missing data.

If included studies data are missing without any description or wrong data, we intend to contact the corresponding author to get an explanation. And consensus will be reached by discussion with a third party (Zhongyu Zhou) if there is a dispute in data extraction.

#### 2.5.3 Assessment of risk of bias and reporting quality of included studies

Two reviewers (Dan Wei and Yang Jiao) will use Cochrane risk-of-bias tool (ROB 2.0 (Centre for Evidence-Based Medicine Odense (CEBMO), Odense, DK)) to evaluate the quality of included studies respectively.^[25]^ The following 5 domains will be used:

1. bias arising from the randomization process,
2. bias due to deviations from intended interventions,
3. bias due to missing outcome data,
4. bias in measurement of the outcome,
5. bias in selection of the reported result.

At last, there is an overall risk of bias of a study after evaluation. Any disagreements about quality of included studies will be solved by discussion with the third reviewer (Fu Dong).

#### 2.5.4 Measurement of treatment effect

In this meta-analysis, statistical analyses will be operated by STATA14.0 software. Dichotomous data will be expressed by the risk ratio (RR) and 95% confidence interval (CI). Continuous data will be expressed by the mean difference (MD) of 95% CI. If different measurement scales are used, standardized mean difference (SMD) will be operated to analyze it.

#### 2.5.5 Dealing with missing data

We will contact the first author or corresponding author to ask for an explanation when data is insufficient. If we receive no response, this study will be moved out of our meta-analysis.

#### 2.5.6 Assessment of heterogeneity

*I*^2^ test is used to evaluate the heterogeneity of included studies. If there is no significant heterogeneity (*I*^2^ < 50%), the fixed-effects model is used. If there is a large heterogeneity (*I*^2^ ≥ 50%), use a random-effects model.

#### 2.5.7 Assessment of publication bias

If included studies>10, a funnel plot analysis will be used to assess publication of bias.

#### 2.5.8 Subgroup analysis

If data is reasonable, we are going to conduct subgroup analysis of different types of groups, the following subgroups we will conduct: different treating time, herbal medicine on patch, different control measures (western medicine, placebo herbal patch, no treatment, acupuncture, moxibustion, etc.)

#### 2.5.9 Sensitivity analysis

Sensitivity analysis can find the source of heterogeneity and confounding factors in the research. When we end up with assessing the quality of included studies. Then we will obtain a stable consolidated result of our study.

#### 2.5.10 Quality of evidence

Quality of evidence will be evaluated by the Grading of Recommendations Assessment, Development, and Evaluation system (GRADE). In this system, evidence quality will be graded by 4 levels (very low, low, moderate, and high) and 5 factors will degrade (study limitations, inconsistency, indirectness, publication bias, and imprecision) level of evidence quality.

## 3 Discussion

AR is a totally tricky health problem that nearly 1 in every 6 American suffered from this disease and generated $2 to $5 billion expenses in global public health system according to published survey.^[26]^ Federally funded national health surveys, such as NHIS and NHANES, point out that it affects 30 to 60 million people in the United States annually, including 10% to 30% of adults and 40% of children.^[27]^ With disturbance of symptoms, patients can do nothing but to seek medicines for help. However, adverse effects of western medicines cannot be ignored. Using oral H1 antihistamines and intranasal corticosteroids spray frequently no doubt makes medicines less effective and the dosage of drugs will be increasing. So how to treat AR without drug resistance has perplexed clinical doctors for a long time. Thus, researchers shift their focus on complementary and alternative therapies, and find some of them indeed reduce the incidence of comorbidities.^[28]^ SAHP, a special acupoint herbal patching mixed acupoint stimulation and time effect, has become a popular therapy to treat AR with prosperity of TCM. Compared to western medicine, SAHP has advantages in safety, low expense, no drug resistance and fewer adverse effects. According to the historical finding about ancient China, the first appearance of SAHP derived from Zhangs Treatise on General Medicine which was wrote by Zhang Lu in Qing dynasty (1636–1912). He insisted that some diseases would occur in winter when yang was insufficient, so doctors use SAHP positively during the summer months on the sanfu days for the reason that this special period has strongest power of yang.^[29]^ In the theory of TCM, this is the principle which is called “treating winter diseases in summer”. Several systematic reviews have proved that SAHP is an effective treatment for chronic diseases, especially in AR.^[30,31]^ Based on theory of meridians, modern biologists have discovered that linkages between qi and blood of organs are expressed by meridians,^[32]^ applying some Chinese herbals on the skin and making it fully absorbed can stimulate reaction of meridians.

With increasing attention to individual health, thousands of people go to medical institutions for SAHP therapy vigorously in summer.^[33]^ Nowadays, an increasing number of clinical studies have reported that SAHP is an effective and safety therapy for treating AR.^[24,34]^ However, systematic review with protocol which use the total effect rate as main outcome has not been published up to now. There are also some limitations on previous studies either meta-analysis without protocol or no secondary outcomes. Therefore, we aim to perform a meta-analysis to assess the effectiveness and safety of SAHP for treating AR. We sincerely wish our research can be a better option therapy for patient with AR, and offer a high quality evidence to explore AR disease.

## Acknowledgments

We thank National Administration of Traditional Chinese Medicine for providing the funding for this research (Award number:2019XZZX-ZJ006), PhD Chengwei Fu of Guangzhou University of Chinese Medicine and MD Tong Wu from Hubei University of Chinese Medicine for their assistance in writing this article and editorial assistance.

## Author contributions

**Conceptualization:** Qiaochu Zhu.

**Data curation:** Aiqun Song, Zhongyu Zhou.

**Formal analysis:** Xiaohui Tian, Yan Wang.

**Investigation:** Qiaochu Zhu, Yangpu Zhang.

**Software:** Dan Wei, Yang Jiao, Fu Dong.

**Supervision:** Aiqun Song, Yangpu Zhang.

**Writing – original draft:** Qiaochu Zhu.

**Writing – review & editing:** Qiaochu Zhu.

## References

[1] Bernstein DI Schwartz G Bernstein JA Allergic rhinitis: mechanisms and treatment. Immunol Allergy Clin North Am 2016;36:261–78. 27083101 10.1016/j.iac.2015.12.004

[2] Greiner AN Hellings PW Rotiroti G Allergic rhinitis. Lancet 2011;378:2112–22. 21783242 10.1016/S0140-6736(11)60130-X

[3] Bernstein JA Allergic and mixed rhinitis: epidemiology and natural history. Allergy Asthma Proc 2010;31:365–9. 20929601 10.2500/aap.2010.31.3380

[4] Meltzer EO Blaiss MS Naclerio RM Burden of allergic rhinitis: allergies in America, Latin America, and Asia-Pacific adult surveys. Allergy Asthma Proc 2012;33: Suppl 1: S113–41. 22981425 10.2500/aap.2012.33.3603

[5] Brozek JL Bousquet J Baena-Cagnani CE Allergic Rhinitis and its Impact on Asthma (ARIA) guidelines: 2010 revision. J Allergy Clin Immunol 2010;126:466–76. 20816182 10.1016/j.jaci.2010.06.047

[6] Meltzer EO Allergic rhinitis: burden of Illness, quality of life, comorbidities, and control. Immunol Allergy Clin North Am 2016;36:235–48. 27083099 10.1016/j.iac.2015.12.002

[7] Meltzer EO Bukstein DA The economic impact of allergic rhinitis and current guidelines for treatment. Ann Allergy Asthma Immunol 2011;106: 2 Suppl: S12–6. 21277528 10.1016/j.anai.2010.10.014

[8] Casale TB Dykewicz MS Clinical implications of the allergic rhinitis-asthma link. Am J Med Sci 2004;327:127–38. 15090751 10.1097/00000441-200403000-00004

[9] Rosenwasser LJ Treatment of allergic rhinitis. Am J Med 2002;113: Suppl 9A: 17S–24S. 12517578 10.1016/s0002-9343(02)01433-x

[10] Ridolo E Montagni M Melli V Pharmacotherapy of allergic rhinitis: current options and future perspectives. Expert Opin Pharmacother 2014;15:73–83. 24219793 10.1517/14656566.2014.860445

[11] Platt M Pharmacotherapy for allergic rhinitis. Int Forum Allergy Rhinol 2014;4: Suppl 2: S35–40. 25182353 10.1002/alr.21381

[12] Pampura AN Papadopoulos NG Spicak V Evidence for clinical safety, efficacy, and parent and physician perceptions of levocetirizine for the treatment of children with allergic disease. Int Arch Allergy Immunol 2011;155:367–78. 21346367 10.1159/000321181

[13] Li XM Complementary and alternative medicine in pediatric allergic disorders. Curr Opin Allergy Clin Immunol 2009;9:161–7. 19295428 10.1097/ACI.0b013e328329226f PMC2770271

[14] Kung YY Chen YC Hwang SJ The prescriptions frequencies and patterns of Chinese herbal medicine for allergic rhinitis in Taiwan. Allergy 2006;61:1316–8. 17002708 10.1111/j.1398-9995.2006.01152.x

[15] Huang TP Liu PH Lien AS A nationwide population-based study of traditional Chinese medicine usage in children in Taiwan. Complement Ther Med 2014;22:500–10. 24906590 10.1016/j.ctim.2014.04.002

[16] Feng S Han M Fan Y Acupuncture for the treatment of allergic rhinitis: a systematic review and meta-analysis. Am J Rhinol Allergy 2015;29:57–62. 25590322 10.2500/ajra.2015.29.4116

[17] Zhou F Yang D Lu JY Characteristics of clinical studies of summer acupoint herbal patching: a bibliometric analysis. BMC Complement Altern Med 2015;15:381. 26492938 10.1186/s12906-015-0905-z PMC4618877

[18] Chen X Lu C Stalsby-Lundborg C Efficacy and Safety of Sanfu Herbal Patch at Acupoints for Persistent Allergic Rhinitis: Study Protocol for a Randomized Controlled Trial. Evid Based Complement Alternat Med 2015;2015:214846. 26300945 10.1155/2015/214846 PMC4537715

[19] Tai CJ Chien LY The treatment of allergies using Sanfujiu: A method of applying Chinese herbal medicine paste to acupoints on three peak summer days. Am J Chin Med 2004;32:967–76. 15673201 10.1142/S0192415X04002569

[20] Chun-lan LWLX Comparative Study of Sanfu Acupuncture and Sanfu Acupoint Application in the Treatment of Allergic Rhinitis. J Clin Acupunct Moxibustion 2017;33:1–5.

[21] Hong-li Z Ming-hui Z Ye-tao H Observations on the efficacy of dog days’ acupoint application in treating allergic rhinitis. Shanghai J Acu-mox 2017;36:588–93.

[22] Huang Q Chen L Chen R Clinical study on treatment of allergic rhinitis of lung qi deficiency and cold type by winter disease and summer treatment. China J Tradit Chin Med Pharmacy 2018;33:2189–93.

[23] Qingde Z A clinical study on treating pediatric allergic rhinitis by the Sanfutian moxibustion plus syndrome differentiation. Clin J Chin Med 2019;11:109–11.

[24] Zhifeng T Clinical observation on treating allergic rhinitis with Sanfu Tie. Clin J Chin Med 2019;11:123–6.

[25] Stewart LA Clarke M Rovers M Preferred reporting items for systematic review and meta-analyses of individual participant data: the PRISMA-IPD statement. JAMA 2015;313:1657–65. 25919529 10.1001/jama.2015.3656

[26] Seidman MD Gurgel RK Lin SY Clinical practice guideline: allergic rhinitis. Otolaryngol Head Neck Surg 2015;152: 1 Suppl: S1–43. 10.1177/0194599814561600 25644617

[27] Wallace DV Dykewicz MS Bernstein DI The diagnosis and management of rhinitis: an updated practice parameter. J Allergy Clin Immunol 2008;122: 2 Suppl: S1–84. 18662584 10.1016/j.jaci.2008.06.003

[28] Fu CW Shu Q Jiao Y A comparison of noninvasive and invasive acupuncture in preventing postoperative nausea and vomiting: a protocol for systematic review and Bayesian network meta-analysis. Medicine (Baltimore) 2020;99:e21544. 32756210 10.1097/MD.0000000000021544 PMC7402802

[29] Wei C Zhang X Li P Acupoint herbal patching during Sanfu Days on reducing frequency of acute asthma attack in children: A systematic review and meta-analysis. Medicine (Baltimore) 2020;99:e18962. 32000423 10.1097/MD.0000000000018962 PMC7004639

[30] Zhou F Yan LJ Yang GY Acupoint herbal patching for allergic rhinitis: a systematic review and meta-analysis of randomised controlled trials. Clin Otolaryngol 2015;40:551–68. 25754265 10.1111/coa.12410

[31] Pang L Zhang H Lu X Preventive and therapeutic effectiveness of Sanfu acupoint herbal patching for chronic obstructive pulmonary disease at stable stages: a systematic review and Meta-analysis. J Tradit Chin Med 2020;40:530–49. 32744021 10.19852/j.cnki.jtcm.2020.04.003

[32] Wang GJ Ayati MH Zhang WB Meridian studies in China: a systematic review. J Acupunct Meridian Stud 2010;3:1–9. 20633509 10.1016/S2005-2901(10)60001-5

[33] Zhou F Wu HJ Zhai JP Who are the users of a traditional Chinese sanfu acupoint herbal patching therapy in China?: a cross-sectional survey. Medicine (Baltimore) 2016;95:e5414. 27930519 10.1097/MD.0000000000005414 PMC5265991

[34] Jiansheng HLHY Clinical trials of acupoint sticking in combination with sichuan pepper prescription on the treatment of allergic rhinitis in the dog days. J Basic Chin Med 2018;24:1465–6.

